# High Prevalence of *qnr* and *aac(6′)-Ib-cr* Genes in Both Water-Borne Environmental Bacteria and Clinical Isolates of *Citrobacter freundii* in China

**DOI:** 10.1264/jsme2.ME11308

**Published:** 2011-12-13

**Authors:** Rong Zhang, Tomoaki Ichijo, Yong-Lu Huang, Jia-Chang Cai, Hong-Wei Zhou, Nobuyasu Yamaguchi, Masao Nasu, Gong-Xiang Chen

**Affiliations:** 1 Second affiliated hospital of Zhejiang University, Zhejiang University, 88 JieFang Rd, Hangzhou 310009, China; 2 Graduate School of Pharmaceutical Sciences, Osaka University, 1–6 Yamadaoka, Suita, Osaka 565–0871, Japan

**Keywords:** *Enterobacteriaceae*, environmental bacteria, quinolone resistance gene

## Abstract

We investigated the prevalence of *qnr* and *aac(6′)-Ib-cr* genes in water-borne environmental bacteria and in clinical isolates of *Enterobacteriaceae*, as well as the subtypes of *qnr*. Environmental bacteria were isolated from surface water samples obtained from 10 different locations in Hangzhou City, and clinical isolates of *Citrobacter freundii* were isolated from several hospitals in four cities in China. *qnrA*, *qnrB*, *qnrS*, and *aac(6′)-Ib-cr* genes were screened using PCR, and the genotypes were analyzed by DNA sequencing. Ten of the 78 Gram-negative bacilli isolated from water samples were *C. freundii* and 80% of these isolates carried the *qnrB* gene. *qnrS1* and *aac(6′)-Ib-cr* genes were detected in two *Escherichia coli* isolates and *qnrS2* was detected in one species, *Aeromonas punctata*. The *qnr* and *aac(6′)-Ib-cr* genes were present in 75 (72.8%) and 12 (11.6%) of 103 clinical isolates of *C. freundii*, respectively. Of the clinical *C. freundii* isolates with the *qnr* gene, 65 isolates (63.1%) carried *qnrB*, but only three (2.9%) and one (1.0%) carried *qnrA1* and *qnrS2*, respectively, while five isolates carried both *qnrA1* and *qnrB*, and one isolate carried both *qnrS1* and *qnrB*. The *qnrB9* gene was the dominant *qnrB* subtype, followed by *qnrB8* and *qnrB6*. Southern hybridization studies indicated that the *qnr* genes are located on different plasmids. Plasmids isolated from both environmental and clinical *C. freundii* isolates appeared to be homogenous.

Quinolones, which have a broad spectrum of antibacterial activity, have been widely used for chemotherapy and have led to increased resistance of bacteria. Resistance to quinolones is mainly due to chromosomally mediated mechanisms, including mutations in quinolone targets (DNA gyrase and topoisomerase IV) and decreased accumulation of quinolones (porin alternation or overexpression of efflux pump systems) (21). The first plasmid-mediated quinolone resistance (PMQR) determinant was identified in *Klebsiella pneumoniae* in 1998 (11). Cloning of the gene identified this determinant as a 657-bp fragment encoding a protein with 218 aminoacid residues, which was named Qnr (more recently termed QnrA) (29); QnrB and QnrS were discovered subsequently (5, 6). Very recently, two novel *qnr* genes, *qnrC* and *qnrD*, were reported (3, 30). In addition to Qnr, two new types of PMQR determinants have been described. The *aac (6′)-Ib-cr* aminoglycoside acetyltransferase gene, whose product is capable of acetylating ciprofloxacin and norfloxacin, was discovered in *qnrA*-positive *Escherichia coli* in 2006 (18). QepA, a plasmid-mediated fluoroquinolone efflux pump, was identified in two clinical isolates of *E. coli*, one from Belgium and one from Japan, in 2007 (14, 33).

Qnr and *aac (6′)-Ib-cr* determinants have now been identified worldwide in many different enterobacterial species (17, 19, 26). These determinants can also be detected in *E. coli* isolates from poultry and swine (35) and in *Enterobacteriaceae* from pets, livestock and poultry (10). To better understand the transfer and prevalence of drug-resistant pathogens and determinants in the human-environment system, we collected bacteria from both water samples and clinical patients, and analyzed the *qnr* and *aac (6′)-Ib-cr* genes. We found that a high percentage of the environmental samples shared the *qnrB* gene in common with the clinical samples.

## Materials and Methods

### Bacterial strains

Water samples were collected from 10 distinct aquatic environments (including West Lake, Qiantang River, Jinghang Grand Canal, Xixi Wetland, Jiefang River, Huajiachi Lake, Jiuxi River, Tiesha River, two fountains in Qingchun Square and the 2nd Affiliated Hospital of Zhejiang University [SAHZU]) in Hangzhou, China during October to November 2008. Five to ten representative sites in each locality were selected for sample collection. We selected those sampling location to represent the main water environments of the city, including artificial fountain, rivers with running water, large volume lakes, and small ponds. Bacteria in water samples (1 L samples) were concentrated by centrifugation and inoculated onto blood-, MacConkey-, and thiosulphate citrate bile salts sucrose (TCBS)-agar plates. Clinical isolates of *Citrobacter* spp. were isolated from SAHZU and collected from several other hospitals in four cities (Beijing, Shanghai, Hangzhou, and Wenzhou) in China during January to December 2008, in a drug resistance surveillance program. All the collected isolates were from sources such as sputum, urine and bodily secretions. All of these isolates were identified using the Vitek System (bioMérieux, Hazelwood, MO, USA).

### Antimicrobial susceptibility testing

The minimal inhibitory concentration (MIC) of ciprofloxacin, levofloxacin and nalidixic acid against bacteria was determined using the agar dilution method according to Clinical and Laboratory Standards Institute (CLSI) recommendations (4). MIC results were determined after incubation at 35°C for 16–20 hours. Muller-Hinton agar was purchased from Oxoid (Hampshire, UK).

### PCR amplification and sequencing

Screening of *qnrA*, *qnrB*, *qnrS* and *aac(6′)-Ib* genes was carried out by PCR amplification using specific primers (8, 20). Colonies were boiled to prepare DNA templates for PCR. The reaction was conducted in a Tpersonal thermal cycler (Whatman Biometra, Goettingen, Germany) as previously described (20). The PCR products were sequenced using an ABI3730 Sequencer (Applied Biosystems, Carlsbad, CA, USA), and the obtained sequences were compared with the sequences deposited in GenBank.

### Transconjugation and transformation studies

For these studies the donors were the *qnr-*carrying strains isolated in this study, while rifampicin-resistant *E. coli* J53 was used as the acceptor. The transconjugated strains were screened on a medium including sulfamethoxazole or levofloxacin. The detailed experimental method was as described by Wang *et al.*(30).

### Southern hybridization

The amplified products of *qnrB* and *qnrS* of the environmental isolates were labeled using the DIG High Primer DNA Labeling and Detection Starter Kit I (Roche, Diagnostics, Mannheim, Germany), according to the manufacturer’s instructions. Plasmid and chromosomal DNA was extracted from the *qnr-*carrying water-borne strains using a kit from Axygen (Axygen Scientific, Union City, NJ, USA). The DNA was trans-blotted to a nylon membrane from ethidium bromide (EB)-free 0.8% agar gel after 1.5 h of electrophoresis, and was then hybridized to the probe using the DIG High Primer DNA Labeling and Detection Starter Kit I according to the manufacturer’s instructions.

## Results

### Bacteria isolated from aquatic environments

Seventy-eight Gram-negative bacilli were isolated from water samples, including 33 *Enterobacteriaceae*, 21 *Aeromonas* spp., ten *Acinetobacter* spp., ten *Pseudomonas* spp., two *Alcaligenes* spp., two *Plesiomonas* spp. and ten *Citrobacter freundii* (Table 1). Gram-negative cocci or Gram-positive bacteria were not obtained in the current study.

### Quinolone susceptibility of environmental and clinical isolates

The MIC_50_ and MIC_90_ of ciprofloxacin against 78 water-borne environmental isolates were ≤0.125 μg mL^−1^ and 16 μg mL^−1^, respectively. The MICs of ciprofloxacin, levofloxacin and nalidixic acid, as well as the MIC_50_/MIC_90_ ratios and the MIC range against each genus are shown in Table 1. The MIC_50_ and MIC_90_ of ciprofloxacin and levofloxacin against the water-borne environmental *C. freundii* were ≤0.125 μg mL^−1^ and 0.25 μg mL^−1^; 0.125 μg mL^−1^ and 0.25 μg mL^−1^, respectively, while those of nalidixic acid were 4 μg mL^−1^ and 8 μg mL^−1^, respectively.

The overall MIC_50_ and MIC_90_ of ciprofloxacin against 103 clinical isolates of *C. freundii* were 2 μg mL^−1^ and 32 μg mL^−1^, respectively. There was little difference in the MIC_50_ and MIC_90_ for samples collected from hospitals in different cities. The MIC_50_ and MIC_90_ against clinical samples from the four cities were 4 μg mL^−1^ and 16 μg mL^−1^, ≤0.125 μg mL^−1^ and 32 μg mL^−1^, 0.5 μg mL^−1^ and 16 μg mL^−1^, and 1 μg mL^−1^ and 32 μg mL^−1^, for samples from Hangzhou, Wenzhou, Shanghai, and Beijing respectively. The MIC_50_ and MIC_90_ of ciprofloxacin against *Citrobacter braakii* were 4 μg/ml and >128 μg mL^−1^, respectively. The MIC of ciprofloxacin against all isolates of *Citrobacter koseri* and *Citrobacter amalonaticus* was ≤0.125 μg mL^−1^.

### Prevalence of the *qnr* and *aac(6′)-Ib-cr* genes in environmental and clinical isolates

Ten of the water-borne environmental isolates were *C. freundii* and 80% of these carried the *qnrB* gene (Table 2). The *qnrS* gene was detected in one *E. coli* and one *Aeromonas* sp. The *aac(6′)-Ib* gene was detected in other *E. coli* strain and another *Aeromonas* sp. The two *Aeromonas* spp. were identified as *Aeromonas punctata* based on the results of sequencing of the *gyrB* gene (32). The *qnrS1* gene in *E. coli* and the *qnrS2* gene in *A. punctata* were identified by sequencing. One of the three isolates that carried the *aac(6′)-Ib* gene carried the *-cr* variant. *E. coli* and *A. punctata* were all isolated from Huajiachi Lake. Three of the eight *C. freundii* isolates that carried the *qnrB* gene were isolated from Huajiachi Lake, two were from West Lake and one each was isolated from the Qiantang River, Jinghang Grand Canal and the Xixi Wetland.

Of the 103 clinical isolates of *C. freundii*, 75 (72.8%) and 12 (11.6%) carried the *qnr* and *aac(6′)-Ib-cr* genes, respectively. *qnrA*-, *qnrB*- and *qnrS*-type alleles were detected in eight (7.8%), 71 (68.9%), and two (1.9%) isolates. Some of these isolates carried two types of *qnr* alleles (Table 2). The rate of *qnr* carriage among *C. freundii* isolates from Beijing, Shanghai, and Wenzhou was a little higher than 50%, while that from Hangzhou was 74.5%. The sequences of the *qnrA* genes of the isolates all matched that of *qnrA1*(12), and the sequences of the *qnrS* genes of the isolates from Hangzhou and Shanghai matched those of *qnrS1* and *qnrS2*(12), respectively. We used three different restriction endonucleases (*Apa*I, *Hind*III and *Xba*I) to digest the two plasmids. The restriction fingerprinting of the plasmids was different, indicating that although the two plasmids are of a similar size, they are different. The common subtypes of *qnrB* detected in 79 water-borne environmental and clinical isolates of *C. freundii* were *qnrB9* (27 isolates), *qnrB8* (16 isolates) (12), *qnrB6* (11 isolates), and *qnrB4* (6 isolates), and other subtypes, including *qnrB10*, *qnrB11*, *qnrB12*, *qnrB13*, *qnrB16*, *qnrB17*, and *qnrB18*, were also detected (7). We compared the *qnrB* sequences of the clinical and environmental strains, but no mutations in the *qnrB* gene were found in genes that originated from either the environmental or clinical strains.

Interestingly, 11 of 12 isolates that carried *aac(6′)-Ib-cr* also carried one or two types of *qnr*. Four isolates carried *qnrA1*, *qnrB8*, and *aac(6′)-Ib-cr*, three isolates carried *qnrA1* and *aac(6′)-Ib-cr*, two isolates carried *qnrB6* and *aac(6′)-Ib-cr*, two isolates carried *qnrB9* and *aac(6′)-Ib-cr*, and one isolate carried only *aac(6′)-Ib-cr*. Of the clinical isolates of other *Citrobacter* spp., two of the seven *C. braakii* isolates carried both *qnrA1* and *aac(6′)-Ib-cr*; one *C. braakii* isolate carried *qnrB11*; one *C. braakii* isolate carried *aac(6′)-Ib-cr*; one of three *C. amalonaticus* isolates carried *qnrB9*. No *qnr* or *aac(6′)-Ib-cr* gene was detected in *C. koseri*.

### Distribution of the MIC of ciprofloxacin against isolates carrying *qnr*- or *aac(6′)-Ib-cr*-genes

**Fig. 1:**
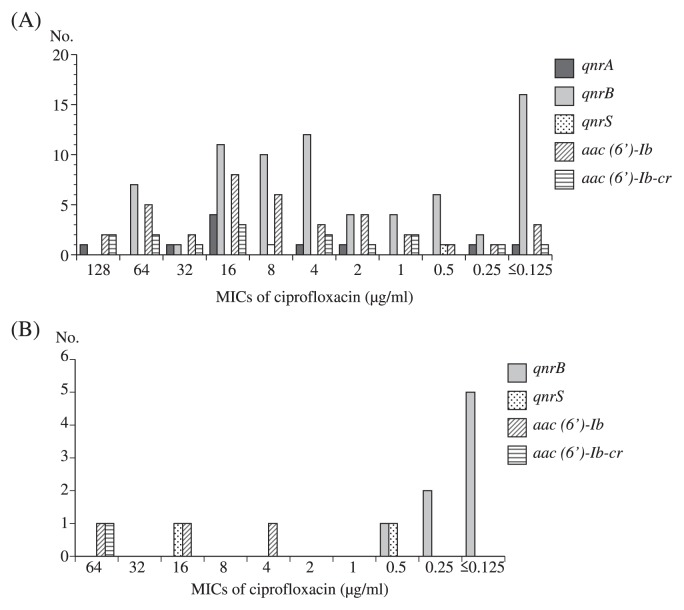
Distribution of MIC of ciprofloxacin among isolates with *qnr* or *aac(6′)-Ib-cr* gene. (A) Clinical isolates. (B) Water-borne environmental isolates.

A wide range of MICs of ciprofloxacin against isolates that carried the *qnr-* or *aac(6′)-Ib-cr-*genes was observed, which varied from ≤0.125 μg mL^−1^ to 128 μg mL^−1^ (Fig. 1). The MIC of ciprofloxacin against most *qnrA1-* or *aac(6′)-Ib-cr*-carrying isolates was ≥0.25 μg mL^−1^, except for one clinical isolate of *C. freundii* that carried *qnrA1*, *qnrB8*, and *aac(6′)-Ib-cr* (MIC ≤0.125 μg mL^−1^). Similarly, the MIC of ciprofloxacin against four *qnrS*-carrying isolates (4/200, 2.0%) was ≥0.5 μg mL^−1^; however, for *qnrB-*carrying isolates, the MIC of ciprofloxacin against 16 of 71 clinical isolates (21.9%), and five of eight water-borne environmental isolates of *C. freundii* (62.5%) was ≤0.125 μg mL^−1^.

### Southern hybridization

**Fig. 2:**
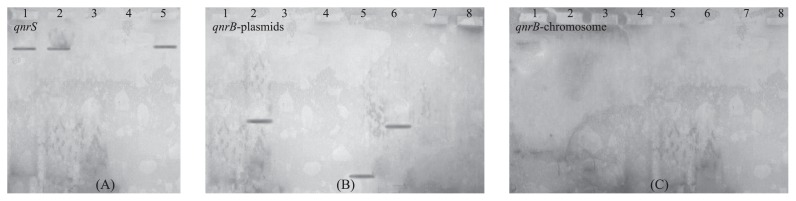
Southern hybridization analysis for determination of the location of *gnr* gene. (A) Southern hybridization analysis of plasmid or chromosomal DNAs targeting *gnrS* gene. Lane 1, Positive control for *qnrS* gene; Lane 2 or 5, plasmid DNAs from environmentally isolated *E. coli* and *A. punctata*, respectively; Lane 3 or 4, chromosomal DNAs from environmentally isolated *E. coli* and *A. punctata*, respectively. (B) Southern hybridization analysis of plasmid DNAs from eight environmentally isolated *C. fruendii* targeting *gnrB* gene. (C) Southern hybridization analysis of chromosomal DNAs from eight environmentally isolated *C. fruendii* targeting *gnrB* gene.

Southern hybridization using a *qnrS* probe identified a specific band (Table 3 and Fig. 2) corresponding to the plasmid DNA of environmental *E. coli* or *Aeromonas* sp. isolates, whereas no band that corresponded to chromosomal DNA was observed, suggesting that the *qnrS* gene is located on the plasmid with an approximate size of 53 kb. Using Southern hybridization we also confirmed that a *qnrB* probe hybridized to plasmid DNA but not to chromosomal DNA of three of the *qnrB*-carrying environmental *C. freundii* isolates. No hybridized band was identified from either the plasmid or the chromosomal DNA of five other *qnrB*-carrying isolates. Since no hybridization was observed for chromosomal DNA, this indicates that *qnrB* genes are not found on the chromosomes. It is possible that lower copy numbers of the *qnrB* gene are located on the plasmids of these strains.

## Discussion

*C. freundii* is one of the normal flora in human and animal intestines; however, it is also an opportunistic pathogen, which can cause diarrhea, septicaemia, meningitis, and brain abscess. It was ranked as No. 13 of the most frequently isolated pathogenic Gram-negative bacteria in 2009 in Zhejiang Province. In this study, the presence of *qnr* and *aac(6′)-Ib-cr* genes in water-borne environmental bacterial isolates was screened by PCR and it was found that the prevalence of *qnrB* in *C. freundii* was as high as 80.0%. The prevalence of such genes in clinical isolates of *C. freundii* was therefore investigated. As expected, the rate of *qnr* and *qnrB* carriage in these isolates was high (72.8% and 63.1%, respectively). Moreover, 11.6% of the clinical isolates of *C. freundii* carried the *aac(6′)-Ib-cr* gene, which was not detected in environmental *C. freundii*.

The prevalence of *qnr* and *aac(6′)-Ib-cr* genes appears to vary considerably in different studies depending on the criteria used to select the bacterial strains. The overall prevalence of *qnr* in *Enterobacteriaceae* has been reported to range from 0.2% to 50%, and *aac(6′)-Ib-cr* may be more prevalent than *qnr*(17, 19). The distribution of *qnr* genes in enterobacterial isolates has been investigated in the UK and Spain (9, 12). The prevalence of *qnr* genes, especially *qnrB*, has also been reported for clinically isolated *K. pneumoniae* and other *Enterobacteriaceae* species in Asian countries (24, 28). In China, *qnr* and *aac(6′)-Ib-cr* genes were detected in 8.0% and 9.9% of extended-spectrum β-lactamase (ESBL)-producing *E. coli* and *K. pneumoniae* isolates, respectively, that were collected from six provinces or districts (8). The prevalence of *qnrA*, *qnrB*, *qnrS*, and *aac(6′)-Ib-cr* genes in *Enterobacter cloacae* isolates from Anhui Province in China was below 10% for all of the genes (31); however, only a few studies have investigated the prevalence of *qnr* and *aac(6′)-Ib-cr* genes in *C. freundii*, possibly due to the relatively low rate of *Citrobacter* sp. isolation in a clinical setting compared with that of *E. coli*, *K. pneumonia*, and *E. cloacae*. A Korean study showed that 53 (38.4%) of 138 AmpC-producing *C. freundii* isolates harbored Qnr determinants (13). Another Korean study detected QnrB determinants in 67.9% of *C. freundii*, 62.5% of *K. pneumoniae*, 15.8% of *E. cloacae*, and 9.4% of *E. coli* isolates that were resistant to nalidixic acid and to at least one extended-spectrum β-lactam (27). In another study, *Enterobacteriaceae* isolates from nine teaching hospitals in China were investigated, and the MIC of ciprofloxacin against these isolates was >0.25 μg mL^−1^. Of the isolates for which the MIC of cefotaxime was >2.0 μg mL^−1^, *qnr* was present in 63.3% of *C. freundii*, 65.5% of *K. pneumoniae*, 65.7% of *E. cloacae*, and 6.5% of *E. coli* isolates. The prevalence of the *aac(6′)-Ib-cr* gene in these four bacterial species was 26.7%, 21.8%, 8.6%, and 16.9%, respectively (34). In our study, the 103 clinical isolates of *C. freundii* investigated were collected without any selection criteria; however, the prevalence of *qnr* and *qnrB* was as high as 72.8% and 68.9%, respectively, which is similar to that of its prevalence in water-borne environmental *C. freundii* (80.0%) (Table 2). Clinical isolates of *C. braakii* displayed a high prevalence of *qnr* and *aac(6′)-Ib-cr* (42.9% for both) (Table 2), which may be because *C. braakii* is a member of the *C. freundii* complex or may be due to the high MIC of ciprofloxacin against these isolates. It was noted that *qnrA1* was always combined with *aac(6′)-Ib-cr*; nine of ten isolates with *qnrA1* also carried *aac(6′)-Ib-cr*. Of the 15 isolates with *aac(6′)-Ib-cr*, 13 isolates carried *qnr*, nine of which were *qnrA1* (Table 2). Six variants of *qnrA*, 19 variants of *qnrB*, and three variants of *qnrS* have been identified worldwide (7). All of the *qnrA* detected in this study were identified as *qnrA1*. Of the four *qnrS*-carrying isolates, two isolates carried *qnrS1* and two isolates carried *qnrS2*. The most common subtypes of the *qnrB* subtype detected in this study were *qnrB9*, *qnrB8* and *qnrB6*, which is quite different from the results of previous studies (13, 27, 34).

The *qnrA* and *qnrS* genes have been shown to originate from water-borne environmental bacteria, *Shewanella algae* and *Vibrio splendidus*, respectively (1, 16). A *qnrS2* gene was recently identified in a water-borne bacterial species, *Aeromonas*, isolated from the River Seine in Paris (2) and from a Swiss lake (15). The *qnrS2* gene has also been detected in a clinical *Aeromonas veronii* isolate (22). In the present study, the same *qnrS2* gene was detected in the same bacterial species (Table 2). This is the first report of an isolate of the *Aeromonas* sp. harboring *qnrS2* outside Europe (Table 2). As shown in Table 2, eight out of ten water-borne *C. fruendii* had the *qnrB* gene (2, 6, 17, 26). These results support the hypothesis that *qnr* genes originated from water-borne bacteria; however, for the majority of *qnrB*-carrying environmental *C. freundii*, the MIC for quinolone is low (Fig. 1B), while for the clinical *qnrB*-carrying environmental *C. freundii*, the MIC is widely distributed, indicating that *qnrB* itself does not contribute to the high level of quinolone resistance, but with the involvement of other mechanisms, such as other *qnr* genes, *gyrA* and *parC*, resistance will be elevated. On the other hand, environmental *C. freundii* carrying *qnrS* and *aac(6′)-Ib-cr* have a higher MIC for quinolone. Such a phenomenon was also observed for the clinical isolates, indicating that those genes contribute to the higher level of quinolone resistance.

Most *qnr* genes have been reported to be located on plasmid DNA (25), while a few are located on chromosomal DNA (23); however, in our effort to determine the localization of these genes, we were unable to produce transconjugants and transformants that carry *qnr* genes. The plasmid carrying the *qnrB* gene may be a non-conjugable plasmid. Our Southern hybridization and endonuclease digestion experiments results clearly indicated that *qnrS1*-encoding plasmid and *qnrS2*-encoding plasmid are heterogenous, and that in at least three out of the eight environmental *C. freundii* strains, *qnrB* is located on plasmid(s) (data not shown); however, no bands corresponding to *qnrB* were detected in either plasmid or chromosomal DNA by Southern hybridization of the other *qnrB*-positive environmental *C. freundii*. We suspect that the gene might be located on the plasmids in these strains with very low abundance.

In summary, there is a high prevalence of plasmid-coded *qnr* and *aac(6′)-Ib-cr* genes. The plasmids isolated from both environmental and clinical *C. freundii* isolates appeared to be homogenous. Further investigations are required to confirm the hypothesis that *C. freundii* play a role as a reservoir of the *qnrB* gene and that the aquatic environment is an important vehicle for the spread of PMQR.

## Figures and Tables

**Table 1:** Species distribution of bacteria isolated from aquatic environments and their MICs of ciprofloxacin, levofloxacin and nalidixic acid (μg mL^−1^)

Strain	No.	Ciprofloxacin		Levofloxacin		Nalidixic acid		Resource^a^

		MIC, or MIC_50_/MIC_90_	MIC Range	MIC, or MIC_50_/MIC_90_	MIC Range	MIC, or MIC_50_/MIC_90_	MIC Range	
*C. freundii*	10	≤0.125/0.25	≤0.125~64	0.125/0.25	≤0.125~1	4/8	2~16	WL, QTR, JHGC, XXW, JFR, HJCL
*E. coli*	9	0.25/64	≤0.125~64	0.03/2	0.03~2	4/>256	1~>256	WL, JHGC, JFR, HJCL, JXR
*K. pneumoniae*	6	≤0.125	≤0.125	0.03/0.06	0.03~0.06	2/4	0.5~4	QTR, XXW, JFR, HJCL, JXR
*E. cloacae*	2	≤0.125	≤0.125	0.03	0.03	2	2	XXW, JXR
*E. aerogenes*	1	≤0.125	—b	0.06	—	2	—	HJCL
*E. intermedius*	1	0.25	—	0.06	—	1	—	HJCL
*Proteus penneri*	1	≤0.125	—	0.05	—	1	—	HJCL
*Pantoea* sp.	1	≤0.125	—	0.06	—	128	—	JFR
*Kluyvera* spp.	2	≤0.125	≤0.125	≤0.015/0.03	≤0.015~0.03	1/4	1~4	QTR, JFR
*Aeromonas* spp.	21	0.25/16	0.125~32	0.25/16	0.125~16	128/>256	64~>256	WL, JHGC, XXW, JFR, HJCL, JXR, TSR, FQCS, FH
Acinetobacter spp.	10	0.5/16	≤0.125~32	0.06/0.5	0.06~1	2/64	0.5~128	QTR, XXW, JXR, TSR, FQCS
*Pseudomonas* spp.	10	≤0.125/1	≤0.125~32	0.06/0.5	0.06~1	1	0.5~1	WL, QTR, JHGH, JFR, HJCL, JXR, TSR
*Alcaligenes* spp.	2	≤0.125	≤0.125~0.25	≤0.015	≤0.015	1	1	WL, TSR
*Plesiomonas shigelloides*	2	≤0.125	≤0.125	≤0.015	≤0.015	1	1	HJCL

a WL, West Lake; QTR, Qiantang River; JHGC, Jinghang Grand Canal; XXW, Xixi Wetland; JFR, Jiefang River; HJCL, Huajiachi Lake; JXR, Jiuxi River; TSR, Tiesha River; FQCS, fountain in Qingchun Square; FH, fountain at 2nd Affiliated Hospital of Zhejiang University

b only one isolate.

**Table 2:** Prevalence of *qnr* and *aac(6′)-Ib-cr* genes in water-borne environmental and clinical bacteria

Resource	Strain		No.	Number of positive isolates (%)						

				*qnrA*	*qnrB*	*qnrS*	*qnrA*+*qnrB*	*qnrS*+*qnrB*	*aac(6′)-Ib*	*aac(6′)-Ib-cr*
*Aquatic environment*	*C. freundii*		10	0	8 (80.0)	0	0	0	0	0
	*E. coli*		9	0	0	1 (11.1)	0	0	1 (11.1)	1 (11.1)
	*Aeromonas* spp.		21	0	0	1 (4.8)	0	0	2 (9.5)	0
	Others		38	0	0	0	0	0	0	0

*Hospital*	*C. freundii*	Hangzhou	51	3 (5.9)	38 (74.5)	0	4 (7.8)	1 (2.0)	25 (49.0)	10 (19.6)
		Wenzhou	14	0	7 (50.0)	0	0	0	2 (14.3)	1 (7.1)
		Shanghai	7	0	4 (57.1)	1 (14.3)	1 (14.3)	0	1 (14.3)	1 (14.3)
		Beijing	31	0	16 (51.6)	0	0	0	5 (16.1)	0
		Total	103	3 (2.9)	65 (63.1)	1 (1.0)	5 (4.8)	1 (1.0)	33 (32.0)	12 (11.6)
	*C. braakii*		7	2 (28.6)	1 (14.3)	0	0	0	4 (57.1)	3 (42.9)
	*C. koseri*		9	0	0	0	0	0	0	0
	*C. amalonaticus*		3	0	1 (33.3)	0	0	0	0	0

**Table 3:** Summary of Southern hybridization of environmental isolates carrying *qnrB* or *qnrS* genes

Strain	No.	Number of positive isolates			

		*qnrB* on chromosome	*qnrB* on plasmid	*qnrS* on chromosome	*qnrS* on plasmid
*qnrB*-positive					
*C. freundii*	8	0	3		
*qnrS*-positive					
*E. coli*	1			0	1
*Aeromonas* sp.	1			0	1
